# A traditional dietary pattern is associated with lower odds of overweight and obesity among preschool children in Lebanon: a cross-sectional study

**DOI:** 10.1007/s00394-017-1574-0

**Published:** 2017-11-10

**Authors:** Lara Nasreddine, Hiba Shatila, Leila Itani, Nahla Hwalla, Lamis Jomaa, Farah Naja

**Affiliations:** 1 Nutrition and Food Sciences Department, Faculty of Agriculture and Food Sciences, American University of Beirut, P.O. BOX 11-0.236, Riad El Solh, Beirut, 11072020 Lebanon; 2 Department of Nutrition and Dietetics, Faculty of Health Sciences, Beirut Arab University, P.O. BOX 11-5020, Riad El Solh, Beirut, 11072809 Lebanon; 3 Nutrition, Obesity and Related Diseases (NORD), Office of Strategic Health Initiatives, American University of Beirut, P.O. Box 11-0236, Riad El Solh, Beirut, Lebanon

**Keywords:** Dietary patterns, Obesity, Overweight, Preschool children, Lebanon

## Abstract

**Purpose:**

The high burden of preschool overweight in the Middle East and North Africa highlights the need for rigorous investigations of its determinants. This study aims at identifying dietary patterns amongst preschoolers in Lebanon and assessing their association with overweight and obesity.

**Methods:**

A national cross-sectional survey was conducted amongst 2–5-year-old children (*n* = 525). Socio-demographic, dietary, lifestyle and anthropometric variables were collected. Dietary patterns were derived by factor analysis. Overweight/obesity was defined based on the World Health Organization 2006 criteria (BMI-for-age *z*-score > + 2).

**Results:**

Two patterns, “Fast Food and Sweets” and “Traditional Lebanese”, were identified. The “Fast Food and Sweets” pattern was characterized by higher consumption of sweetened beverages, fast foods, salty snacks and sweets. The “Traditional Lebanese” was driven by higher intakes of cereals, dairy products, fruits and vegetables. Children belonging to the 3rd tertile of the Traditional pattern scores had significantly lower odds of overweight/obesity compared to the 1st tertile (OR 0.33; 95% CI 0.11, 0.97). Higher maternal education and higher frequency of eating with family predicted adherence to the traditional pattern, while the presence of a household helper was a negative determinant. Adherence to the Fast Food and Sweets pattern was positively associated with the child’s age, and negatively associated with female gender and maternal education.

**Conclusions:**

The “Traditional Lebanese” pattern was associated with decreased risk of preschool overweight. Policies aiming at re-anchoring this traditional dietary pattern in contemporary lifestyles may be developed as potential preventive strategies against overweight in this age group.

## Introduction

Pediatric overweight is recognized as “one of the most serious public health challenges” of our era, with available evidence documenting alarming secular increases in its worldwide prevalence [1–3]. These increases have been noted as early as the preschool years. Available estimates suggest that the highest rates of preschool overweight and obesity are observed in countries of the Middle East and North Africa (MENA) region, ranging between 14.7 and 17% in 2010 [2]. By year 2020 the prevalence of preschool overweight and obesity is expected to exceed 25% in this region, underscoring the magnitude and health implications of this problem. Excess body weight in children may exert negative physical and psychological effects that tend to appear early in life and track into adulthood [4–6]. Cardiometabolic abnormalities such as dyslipidemia, elevated blood pressure, impaired glucose homeostasis and metabolic syndrome are amongst the well-documented short-term health consequences of pediatric obesity [7]. Childhood obesity tends to persist into adulthood, and is often associated, on the long-term, with increased risk for chronic diseases such as type 2 diabetes and cardiovascular diseases [8, 9].

Excess body weight results from a complex interplay between genetic, social, environmental and behavioral factors, including diet, lifestyle and physical activity [10]. High-energy intakes, frequent consumption of fatty foods and sweetened beverages, as well as sedentary behavior have been reported amongst the key contributors to pediatric overweight [4, 10]. Studies focusing on diet as a risk factor for preschool overweight have, however, provided inconsistent evidence [11–14]. This may be partially due to the adoption of traditional methods in nutritional epidemiology, whereby most of the studies examining the association between diet and obesity have investigated the intakes of individual nutrients, foods or food groups [11–14]. Acknowledging the complex and rich array of interactions between the various nutrients, there has been a growing concern that the overall pattern of dietary intakes should be appraised when investigating the association between nutrition and chronic conditions such as obesity [15]. In this context, dietary pattern analysis was proposed as a holistic approach that investigates the joint effects of multiple dietary components on obesity risk [16, 17]. Few studies have examined dietary patterns amongst preschoolers and their association with the risk of overweight or obesity [18–22]. A dietary pattern rich in French fries and sweetened beverages and low in dairy products and water was found to be associated with increased risk of overweight and obesity amongst 3–6-year-old French children [18]. Similarly, Manios et al. showed that an “unhealthy dietary pattern”, characterized by increased consumption of sweets and red meat, was associated with higher risk of obesity amongst 1–5-year-old Greek children [19]. It is important to note that the studies that have previously investigated dietary patterns amongst preschoolers have been mostly conducted in developed countries and as such their findings may not be applicable to low- and middle-income countries, given the context-specific nature of dietary patterns [18, 19, 23, 24]. This may be particularly true for countries of the MENA region, that are currently undergoing the nutrition transition, with its characteristic shifts in dietary habits, food consumption patterns and body composition [25].

In Lebanon, a small country of the MENA region, recent studies have suggested a worrisome increasing trend in the prevalence of childhood obesity over the past decade [26]. The increasing burden of pediatric obesity, paired with its public health implications highlights the need for rigorous investigations of its determinants and associated factors. This study is based on a national survey conducted amongst children under the age of five in Lebanon, which has shown that 9% of Lebanese preschoolers are overweight or obese, and which has reported a positive relationship between dietary fat intake and overweight in this age group [27]. To examine the association between diet and preschool overweight in a more holistic approach, this study aims at (1) deriving and characterizing dietary patterns among Lebanese preschoolers using factor analysis; (2) examining the association of these patterns with socio-demographic and lifestyle characteristics; and (3) evaluating the association of these patterns with overweight and obesity in this age group. By providing further insight into diet–obesity associations in the preschool years, an age that is identified as a critical period for preventive interventions [6], findings of this study could foster the development of effective preventive strategies and policies aiming at curbing the obesity epidemic in Lebanon and other countries of the MENA region.

## Methods

### Study population

**Fig. 1:**
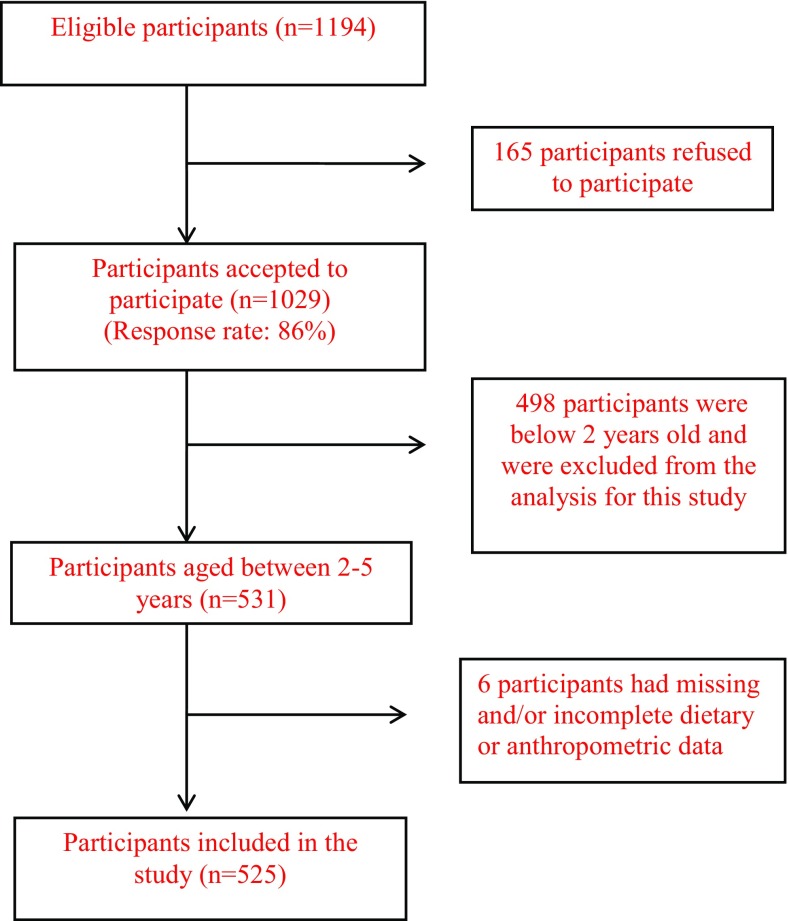
Flowchart describing subjects’ recruitment and participation in the study

The present study was conducted using data from the national survey, “Early Life Nutrition and Health”, conducted in Lebanon between September 2011 and August 2012 [27]. The survey included a representative sample of Lebanese children (0–5 years) and their mothers. The primary sampling unit in the survey was the household. The selection of households followed a stratified cluster sampling strategy, whereby the strata were the six Lebanese governorates and the clusters were selected further at the level of districts. In each district, households were selected based on a probability proportional to size approach, with a higher number of participating households being drawn from more populous districts; households’ selection was conducted using systematic sampling. To be eligible, households ought to include a mother and a child aged 5 years or below. Of the 1194 eligible households that were contacted, 1029 participated in the survey (response rate 86%). Refusal to participate was mainly related to time constraint, child being sick at the time of the interview, absence of husband’s consent or lack of interest in the study. Children and their mothers were not included if children were of non-Lebanese nationality, born preterm (< 37 weeks), or suffered from any chronic illness, inborn errors of metabolism, or physical malformations that may alter dietary intake or body composition [27]. Children who were reported as being ill during the past 24 h (i.e. on the day that would be recalled for dietary intake) were also not included. For the present study, data pertinent to preschoolers aged between 2 and 5 years were considered (*n* = 531). Survey participants who had incomplete dietary or anthropometric data were excluded from the analysis (*n* = 6), hence the total number of subjects in this study was *n* = 525. Figure 1 represents the flowchart depicting subjects’ recruitment and participation in this study (Fig. 1).

Data collection took place at the participants’ house, and was performed by trained research nutritionists through face to face interviews with the mothers, and using an age-specific multi-component questionnaire [27]. Details about the questionnaire development are shown elsewhere [27]. This study was conducted according to the guidelines laid down in the Declaration of Helsinki and the study protocol and procedures were approved by the Institutional Research Board, American University of Beirut (Protocol number NUT.LN.13). Written informed consent was obtained from all mothers prior to participation in the study.

### Data collection

Information about socio-demographic and lifestyle characteristics of study participants was collected, and included: age of the mother and child (in years), sex of the child, marital status of the mother, mother’s and father’s education levels and employment status, presence of a paid helper at home. Information on the type of school the child attends (private vs public) was also obtained since the formal age of schooling is 3 years in Lebanon [27, 28]. The child’s eating habits were also assessed, including the weekly frequency of breakfast and snack consumption, eating the same meal with the family, eating in front of the television (TV) and eating out.

### Anthropometric assessment

Anthropometric characteristics were measured, including weight and height of both mothers and children. Weight and height were not collected from women who were pregnant at the time of the interview (*n* = 42), due to the limitations of body mass index (BMI) use during pregnancy. Body weight was measured to the nearest 0.1 kg with the participant in light indoor clothing and with bare feet or stockings, using a standard clinical scale (Seca balance, model 11770 Germany). Height measurements were obtained without shoes, using a stadiometer. All measurements of weight and height were taken twice and the average values were used in the analysis. BMI was calculated as the ratio of weight (kg) to the square of height (m) [29]. Overweight and obesity among children was assessed using the World Health Organization (WHO) 2006 criteria based on sex- and age-specific BMI *z*-scores [+ 1 < BMI-for-age *z*-score ≤ + 2 (at risk of overweight), + 2 < BMI-for-age *z*-score ≤ + 3 (overweight) and BMI-for-age *z*-score > + 3 (obesity)]. WHO AnthroPlus software was used to derive these* z*-scores.

### Dietary intake of children

Trained dietitians carried out the dietary intake assessment of participating children using the USDA 5-step Multiple Pass 24-Hour Dietary Recall approach (MPR 24-HR), with mothers as proxies [30]. In case another caretaker shared the responsibility of feeding the child, the mother directly consulted with him/her for additional information pertinent to the dietary interview. The MPR 24-HR approach has been shown to reduce the limitations of 24-HRs [31], with its specific steps including: (1) quick food list recall, (2) forgotten food list probe, (3) time and occasion at which foods were consumed, (4) detailed overall cycle and (5) final probe review of the foods consumed. The 24 HR data were analyzed using the Nutritionist Pro software (version 5.1.0, 2014, First Data Bank, Nutritionist Pro, Axxya Systems, San Bruno, CA) and total energy intake estimates were derived. For the analysis of composite, mixed and traditional dishes, standardized recipes were added to the Nutritionist Pro software using single food items. For the analysis, the USDA database was selected (SR 24, published September 2011) within the Nutritionist Pro software.

### Derivation of dietary patterns

Factor analysis (FA) was used to derive dietary patterns in the study population using a total of 15 foods/food groups. The food groups consisted of food items put together based on similarities in ingredients, nutrient profile and/or culinary usage (See Table 5 in Appendix). Before running the FA, the correlation matrix between the 15 food groups was examined using Bartlett test of sphericity and a Kaiser–Meyer–Olkin test (KMO). Both of these tests indicated the suitability of the data for FA. Factors were retained based on: (1) the Kaiser criterion (Eigenvalues > 1), (2) inflection point of the scree plot (retaining factors above the elbow, or break in the plot) (3) and the interpretability of factors [32]. Factor loadings indicated the strength and direction of the association between the patterns and food groups. The derived dietary patterns were characterized and labeled based on food groups that had a factor loading greater than 0.25 [33]. For every child, a score for each dietary pattern was calculated by summing the standardized values of the food items weighted by their scoring coefficient. These scores indicated the degree to which each subject’s diet adhered to the identified pattern.

### Statistical analysis

Sociodemographic as well as lifestyle characteristics of the study population were described using frequencies and percentages as well as means and standard deviations (SD) for categorical and continuous variables, respectively. Simple as well as multiple logistic regression were used to examine the association between socio-demographic and lifestyle characteristics with overweight and obesity. Pearson’s correlation was used to examine the associations of the identified dietary patterns with energy and energy-adjusted nutrient intakes (nutrient intakes were adjusted for energy by the residual method [34]). The association between the identified dietary patterns and overweight and obesity was examined using multiple logistic regression models. In these models, the dependent variable was normal vs overweight or obese and the independent variable was the tertile distribution of the specific pattern. The logistic regression models were adjusted for socio-demographic and lifestyle variables that were found to be significantly associated with overweight and obesity, in addition to age and sex of the child. To examine the socio-demographic and lifestyle correlates of the dietary patterns, multiple logistic regression analyses were used. In these regressions models, adherence to each pattern was the dependent variable (adherence was defined as belonging to the 2nd or 3rd tertile of the pattern score distribution). Variables were put in the model in order of strength of their association with the outcome variable as per the bivariate analysis and according to their reported importance in predicting patterns of dietary intake in the literature. The effect of each variable on the model was assessed and this variable was kept in if it significantly contributed to a better fit of the model. Tests for linearity (Tolerance > 0.4) of the covariates included in the regression models were performed. Normality of the residuals was assessed by the histogram of standardized residuals and normal probability plot in all regression models. Data entry was carried out using Statistical Package for Social Sciences 22.0 (SPSS for Windows, 2013, Chicago: SPSS Inc.). *P* value less than 0.05 was considered statistically significant.

## Results

Of children participating in this study, 48 (9.1%) were either overweight or obese [overweight *n* = 34 (6.4%), obese *n* = 14 (2.6%)]. The association of socio-demographic and lifestyle characteristics with overweight/obesity in the study population is described in Table 1. Results of the multiple logistic regression showed that a higher mother’s BMI was significantly associated with odds of overweight/obesity, whereby a unit increase in the mother’s BMI led to 9% increase in the child odds of overweight/obesity. In addition, a higher father’s education level and presence of a helper at home were associated with greater odds of overweight/obesity. On the other hand, a higher weekly frequency of consuming meals with the family was associated with a lower odd of overweight/obesity (Table 1). Table 2 displays the factor loading matrix which resulted from the FA. In the study population, two major dietary patterns were derived, the “Fast Food and Sweets” and the “Traditional Lebanese” patterns. Together these two patterns explained 20.81% of the total variance in dietary intakes in the study population. The Fast Food and Sweets was driven by foods/food groups such as sweetened beverages (0.6), fast foods (0.53), salty snacks (0.43) and sweets (0.41). On the other hand, the Traditional Lebanese pattern was characterized by higher intakes of breads and cereals (0.57), dairy products (0.53), fruits and vegetables (0.34) and traditional Lebanese mixed dishes (0.23) (Table 2). A further analysis was conducted to examine the daily dietary intake, as assessed by number of servings, of the various foods/food groups by tertiles of pattern scores. The results of this analysis are presented in See Table 6 in Appendix.

**Table 1:** Sociodemographic characteristics and odds of overweight and obesity among Lebanese children 2–5 years old (*n* = 525)

Variables^a^	Normal *n* = 477	Overweight/obesity *n* = 48	aOR ^b^ (95% CI)
Child’s age (years)	3.32 ± 0.88	3.39 ± 0.74	0.84 (0.54–1.31)
Child’s sex			
Male	255 (53.5)	26 (54.2)	1
Female	222 (46.5)	22 (45.8)	1.24 (0.60–2.60)
Mother’s BMI (kg/m^2^)^c^	26.59 ± 5.17	17.86 ± 5.27	**1.09 (1.02–1.16)**
Mother’s age	32.69 ± 5.92	33.73 ± 6.44	1.04 (0.97–1.10)
Mother’s education level			
Primary or less	98 (20.5)	3 (6.3)	1
Up to high school	292 (61.2)	31 (64.9)	1.35 (0.31–5.9)
University	87 (18.2)	14 (29.2)	1.18 (0.21–6.61)
Marital status of the mother			
Not married	9 (1.9)	2 (4.2)	1
Married	468 (98.1)	46 (95.8)	0.28 (0.01–6.12)
Mother’s employment status			
Housewife	409 (85.7)	38 (79.2)	1
Employed	68 (14.3)	10 (20.8)	0.53 (0.19–1.50)
Father’s education			
Primary or less	114 (24.3)	2 (4.3)	1
Intermediate, high school or technical diploma	290 (61.8)	34 (72.3)	**3.79 (0.76–18.84)**
University	65 (13.9)	11 (23.4)	**6.01 (1.02–36.15)**
Father’s employment			
Unemployed	31 (6.5)	4 (8.3)	1
Employed	446 (93.5)	44 (91.7)	0.41 (0.09–1.77)
Presence of a helper at the household			
No	406 (85.7)	33 (68.8)	1
Yes	68 (14.3)	15 (31.3)	**2.77 (1.13–6.76)**
Type of school attended by the child			
Public	102 (27.1)	4 (10.0)	1
Private	274 (72.9)	36 (90.0)	3.47 (0.89–13.48)
Breakfast consumption^d^	6.76 ± 1.68	6.69 ± 1.19	0.92 (0.72–1.17)
Snack consumption^d^	4.62 ± 5.59^f^	6.81 ± 8.97^g^	1.14 (0.96–1.30)
Eating same meal with the family^d^	10.93 ± 6.29^f^	8.99 ± 5.57^g^	**0.93 (0.87–0.98)**
Eating any meal at TV^d^	8.05 ± 7.75^f^	10.64 ± 10.86^g^	0.98 (0.87–1.11)
Eating out^d^	1.68 ± 1.75	1.59 ± 1.61	1.04 (0.83–1.29)

Bold values indicate significant at *p* < 0.05

^a^Categorical variables are expressed as *n*(%), continuous variables are expressed as mean ± SD

^b^aOR were derived from a multiple logistic regression analysis where all variables presented in the table were included in the model, bolded numbers are significant at *p* < 0.05

^c^The number of mothers included in this variable is 483, after exclusion of pregnant women (*n* = 42)

^d^Frequency per week

^f,g^Different superscripts indicate significant difference based on *t* test analysis

**Table 2:** Factor loadings of the identified dietary patterns among Lebanese children 2–5 years old (*n* = 525)

Food group	Dietary patterns	
	Fast food and sweets pattern	Traditional Lebanese pattern
Sweetened beverage	**0.60**	− 0.280
Fast food	**0.53**	–
Salty snacks	**0.43**	− 0.15
Sweets	**0.41**	0.12
Meat, fish, poultry ,eggs	**0.33**	0.19
Lebanese traditional dishes	− 0.29	0.23
Condiments	0.29	0.02
Bread and cereals	0.25	**0.57**
Dairy products	− 0.12	**0.53**
Pizza and pies	0.13	− **0.49**
Tea	**0.32**	**0.42**
Fruits and vegetables	–	**0.34**
Added fats and oils	**0.31**	**0.32**
Starchy vegetables and legumes	–	0.25
Nuts and seeds	–	0.19
Percent variance explained	10.46%	10.35%

Loadings < |0.1| were deleted for simplicity

Loadings greater than |0.3| are in bold

The results of the Person’s correlation showed higher correlation coefficients for the association of the Fast Food/Dessert pattern scores with energy, carbohydrates, fats and sugars as compared to those of Traditional Lebanese pattern (energy: *r* = 0.53 vs 0.26; carbohydrates: *r* = 0.17 vs 0.12; fats: − 0.09 vs − 0.20; sugars: 0.17 vs − 0.15 for the Fast Food/Dessert and Traditional Lebanese patterns, respectively). On the other hand, the score of the Fast Food/Dessert pattern had lower correlation coefficients with proteins, fiber and calcium as compared to the Traditional Lebanese pattern (proteins: *r* = − 0.19 vs 0.15, fiber: *r* = − 0.20 vs 0.16; calcium: − 0.30 vs 0.04 for the Fast Food/Dessert and Traditional Lebanese patterns, respectively). All reported correlation coefficients were significant at *p* < 0.05.

The results of the multiple logistic regression describing the association between adherence to each of the derived patterns and the odds of overweight/obesity in the study sample are shown in Table 3. Of the two patterns, only the Traditional Lebanese was significantly associated with overweight/obesity, whereby children belonging to the highest tertile of the Traditional Lebanese pattern were less likely to be overweight/obese compared to those in the lowest tertile [(odds ratio) OR 0.33, 95% (confidence interval) CI 0.11, 0.97] (Table 3).

**Table 3:** Association of the identified dietary patterns with overweight and obesity among Lebanese children 2–5 years old (*n* = 525)

Tertiles of pattern scores	Odds of overweight/obesity	
	Fast food and sweets pattern	Traditional Lebanese pattern
1	1	1
2	0.86 (0.32–2.32)	0.70 (0.25–1.96)
3	0.82 (0.27–2.53)	**0.33 (0.11–0.97)**

Model adjusted for child age, child sex, mother’s BMI, mother’s education, father’s education, father’s employment, presence of helper in the house, type of school attended, eating the same meal with the family and eating any mean at TV

Numbers in bold are significant at *p* < 0.05

Table 4 describes the sociodemographic and lifestyle correlates of adherence to the identified dietary patterns as identified by multiple logistic regression. Older children were more likely to adhere to the Fast Food and Sweets pattern (OR: 1.31, 95% CI 1.01, 1.71). On the other hand, girls had lower odds of adherence to this pattern (OR: 0.53, 95% CI 0.33, 0.83). While higher education levels of the mother were associated with lower adherence to the Fast Foods and Sweets pattern, children of mothers with a university level of education had three times the odds of adhering to the Traditional Lebanese pattern compared to those whose mothers had a primary or less education level. Furthermore, the presence of helper at home was associated with 45% less odds of adherence to the Traditional Lebanese pattern (OR: 0.55, 95% CI 0.25, 0.85). A higher weekly frequency of meals consumption with the family was also associated with a higher adherence to the Traditional Lebanese pattern (OR 1.11, 95% CI 1.01, 1.15) (Table 4).

**Table 4:** Sociodemographic and lifestyle determinants of adherence to the identified patterns among Lebanese children 2–5 years old (*n* = 525)

Variable	Fast food and sweets		Traditional Lebanese	
	OR	95% CI	OR	95% CI
Child’s age (years)	**1.31**	**1.01, 1.71**	1.19	0.92, 1.55
Child’s sex				
Male	1		1	
Female	**0.53**	**0.33, 0.83**	0.68	0.43, 1.06
Mother’s BMI (kg/m^2^)	1.01	0.969, 1.06	0.99	0.95, 1.04
Mother’s education level				
Primary or less	1		1	
Up to high school	**0.35**	**0.17, 0.73**	1.62	0.89, 2.95
University	**0.20**	**0.08, 0.51**	**3.22**	**1.35, 7.72**
Marital status of the mother				
Not married	1		1	
Married	0.77	0.17, 3.40	1.11	0.26, 4.67
Mother’s employment status				
Housewife	1		1	
Employed	0.53	0.27, 1.02	0.82	0.41, 1.64
Presence of a paid helper at home				
No				
Yes	1.19	0.64, 2.19	0.55	**0.25, 0.85**
Type of school attended by the child				
Public	1		1	
Private	0.57	0.32, 1.03	1.09	0.62, 1.95
Breakfast consumption^a^	1.07	0.91, 1.24	1.02	0.89, 1.17
Snack consumption^a^	1.01	0.91, 1.11	1.04	0.94, 1.14
Eating same meal with family^a^	0.99	0.96, 1.04	**1.11**	**1.01, 1.15**
Eating any meal at TV^a^	1.01	0.94, 1.09	0.96	0.89, 1.03
Eating out^a^	1.05	0.91, 1.21	0.84	0.74, 0.96

Adherence was defined as belonging to the 2nd or 3rd tertile of the pattern score distribution

Numbers in bold are significant at *p* < 0.05

^a^Frequency per week

## Discussion

The present study reports the results of the first national investigation of dietary patterns among Lebanese preschoolers and their association with overweight, lifestyle and socio-demographic factors. Two major dietary patterns were characterized in this population group: (1) the “Fast Food and Sweets” and (2) the “Traditional Lebanese” patterns. Children belonging to the highest tertile of the Traditional Lebanese pattern were less likely to be overweight/obese compared to those in the lowest tertile of this pattern. Together, the identified patterns explained approximately 21% of the total variance in dietary intakes amongst Lebanese 2–5-year-old children, which is in the range of what has been reported in other studies investigating dietary patterns among preschoolers [19, 35–37].

The Fast Food and Sweets pattern, which was characterized by a high consumption of sweetened beverages, fast foods, salty snacks, sweets, and animal foods shares many of the characteristics of the Western dietary pattern that was previously identified amongst Lebanese adolescents [38] and adults [39, 40]. Similarly, the traditional Lebanese pattern, which builds on higher intakes of breads and cereals, dairy products, fruits and vegetables and traditional Lebanese mixed dishes, is similar to the Traditional pattern reported in previous studies conducted amongst adolescents and adults in the country [38–40]. In agreement with our findings, Lioret et al. and Manios et al. have also identified two dietary patterns amongst French and Greek preschoolers, respectively, with the identified patterns being similar to those derived in the present study, even though the specific foods belonging to each pattern may vary in their respective level of contribution [18, 19]. Other studies have, however, characterized more than two patterns amongst preschoolers. For instance, Wall et al. identified the “Junk”, the “Healthy” and the “Traditional” patterns amongst preschoolers in New Zealand, while North et al. identified the “Junk”, the “Health Conscious” and the “Traditional” patterns amongst 4-year-old children in the UK [23, 24]. In our study, a third “Healthy” pattern was not identified, which may be due to the fact that the Traditional Lebanese pattern, which is a variant of the Mediterranean diet [41], shares many of the characteristics of what is known as a “Healthy” pattern, since it builds on fruits, vegetables and cereals, while being low in sweets and fast food. It is, therefore, not surprising that, in this study, the Traditional Lebanese pattern was found to be protective against preschool overweight and obesity. In fact, the lower consumption levels of energy dense foods such as sweets and fast foods may be associated with lower intakes of energy, fat and sugar, thus potentially contributing to energy balance and decreased adiposity risk [42]. Similarly, the higher consumption of fruits, vegetables and dairy products within the Traditional Lebanese pattern may increase the intakes of several nutrients and phytochemicals, including fibre and calcium (Ca) [39]. By decreasing dietary energy density, increasing satiety, buffering insulin fluctuations, and increasing the production of short-chain fatty acids, dietary fibre may pay a role in appetite and weight regulation [43]. Similarly, calcium has been suggested to play a role in body weight regulation. Based on a retrospective analysis of several studies, Heaney et al. (2002) proposed that a daily increase of 300 mg of Ca, or approximately one dairy serving, was associated with a yearly reduction of approximately 1 kg of body fat in youth [44]. The Traditional Lebanese pattern also builds on the utilization of olive oil as the main source of added fat [41], thus increasing the intakes of mono-unsaturated fats, which have been suggested to modulate appetite and adiposity through their effects on body fat oxidation and insulin sensitivity [45, 46].

The results of the present study showed that a higher educational level of the mother was independently associated with higher adherence to the Traditional Lebanese dietary pattern among preschoolers and with lower adherence to the Fast Food and Sweets pattern. Other studies targeting preschoolers have also identified maternal education as a significant determinant of dietary patterns in children of this age group [20, 35]. Lower education amongst mothers predicted higher adherence to the “Processed and Fast Food” dietary pattern amongst French preschoolers and to the “Snacky” pattern amongst Greek under five children [20, 35]. Conversely, North et al. showed that higher maternal education was associated with higher adherence to the “Healthy pattern” among 3-year-old British children. As maternal education is an important determinant of nutrition knowledge [47], it may be possible that mothers with low education levels are not familiar with the importance of healthful diets for their child’s normal development or with the evidence linking the child’s nutritional status with chronic disease risk [19]. Taken together, these findings highlight the role of maternal educational as an important modulator of the family’s environment and eating habits, while also upholding the conclusions reported by Darmon and Drewnowski, denoting that higher quality diets are usually consumed by better educated individuals [48]. The observed positive association between maternal education and adherence to the Traditional dietary pattern may seem contradictory to the fact that maternal education was also found to be significantly associated with a higher risk of preschool overweight in this study. In this context, it is important to note that maternal education may be reflective of a higher socioeconomic status (SES) of the household, suggesting an increased risk of preschool overweight with higher SES [27]. In line with this hypothesis, all the other SES indicators (paternal education; presence of paid helper; type of school) adopted in this study were also found to be associated with a higher risk of overweight amongst Lebanese preschoolers [27]. These findings are in agreement with reports proposing that, in developing countries, childhood obesity is essentially a problem of the rich [49, 50].

Of interest, this study showed that the presence of a household paid helper was significantly negatively associated with adherence to the Traditional Lebanese pattern. It is worth noting that, in Lebanon, the responsibility of child feeding is often delegated to or shared with the household helper. In contrast to parents who tend to play a more active role in guiding the child’s eating behavior, household helpers, who are usually employed for housework as well as child care, may not be able to spend as much time and effort on emphasizing dietary recommendations and promoting the consumption of healthier food choices [51, 52]. In this study, eating the same meal with the family was also found to be associated with higher adherence to the Traditional Lebanese pattern, further underscoring the important role that parents may play in enhancing the quality of the child’s diet and nutritional status [52, 53].

Gender differentials were observed in terms of adherence to the Fast Food and Sweets pattern, with girls being 50% less likely to adhere to this pattern compared to boys. This is in agreement with findings observed amongst French and British preschoolers, where boys had higher adherence to the “Fast Food” or “Junk” dietary pattern, compared to girls [24, 35]. These results may suggest that parenting interactions with respect to food and eating may differ between gender, while also implying that boys and girls may respond differently to parental, and specifically maternal control attempts [54]. It has been proposed that mother’s own dietary restraint and conscious effort to restrict her intakes may predict her attempts to restrict her daughter’s food consumption [54, 55]. The results of this study have also shown that adherence to the Fast Food and Sweets pattern significantly increased with the age of the child. These findings, which are in line with those reported amongst Korean preschoolers [22], may be due to the potentially higher exposure of older children to fast foods, sweets and sweetened beverages, which may in turn shape their food preferences and practices [54].

The strengths of the study include its national representativeness [56, 57], the use of a culture and age-specific questionnaire in data collection and the measurements of anthropometric characteristics instead of relying on self-reporting. The findings of this study should, however, be considered in light of the following limitations. First, the cross-sectional design of the study allows to investigate associations rather than assess causal relationships. Second, food consumption data were based on one 24-HR, which may not be representative of dietary intakes at the individual level. However, despite its potential limitations such as reliance on memory and day-to-day variation, the 24-HR may provide accurate estimates of food intake at the population level [58]. In the present study, dietary intake was assessed by the MPR 24-HR approach, the validity of which has been well established in adults. The evidence among children is less conclusive; however, few reports are showing that this method may also provide accurate estimates of food consumption in children [59, 60]. In addition, the recalls were performed by research nutritionists who underwent extensive training prior to data collection to reduce interviewer errors. Similarly, inter-observer measurement error in anthropometric assessment was minimized by intensive training and follow-up to maintain quality of measurement among all research nutritionists. Furthermore, acknowledging that factor analysis is a data-driven method which tends to define population-specific patterns, it may be argued that this study’s results are likely to represent patterns that are, at least in some aspects, specific to the Lebanese preschool population. In addition, the use of factor analysis requires several arbitrary assumptions relevant to the selection of food groups, the number of retained factors and their labeling [61]. The food groupings that were adopted in this study were comparable with those performed in previous investigations amongst Lebanese adults [39, 40, 62] and adolescents [38]. Lastly, in this study, there was no information about a few factors that may also affect overweight/obesity among children such as maternal pre-pregnancy BMI, gestational weight gain and breastfeeding as well as the level of physical activity of the child [63, 64].

In conclusion, the present study identified two main dietary patterns among the Lebanese preschoolers: the “Traditional Lebanese” and the “Fast Food and Sweets”, with the Traditional Lebanese pattern being associated with a lower risk of overweight in the study population. These findings should catalyze the development of culture-specific interventions aiming at reducing the rates of childhood overweight and obesity, which have been shown to be on the rise in Lebanon [26]. Interventions fostering the adoption of the Traditional Lebanese dietary pattern, characterized by high intake of fruits, vegetables, cereals and dairy products, while being low in energy-dense foods such as fast food, sweets and sweetened beverages, should be developed. In fact, the delivery of dietary guidance based on a dietary pattern approach is usually clearer and easier to understand and interpret compared with nutrient-based approaches, and this may be particularly true for young children and their caregivers [65]. Acknowledging that healthy eating patterns initiated early in life may have immediate nutritional benefits, while also possibly tracking throughout adulthood, interventions aimed at encouraging and establishing healthy dietary behaviors and food skills in early childhood may play a role in modulating the individual’s lifetime risk for diet-related diseases [66].

## Appendix

See Tables 5 and 6.

**Table 5:** Food groupings based on culinary usage and nutrient content

Food group	Food items
Sweetened beverages	All sweetened carbonated soda and sugar-sweetened fruit juices (regular soda, sugar preserved condensed juices, packed juice, chocolate powder, hot chocolate)
Fast food	French fries, sandwich shawarma, hamburger, falafel, nuggets, broasted chicken, escalope, sandwiches
Salty snacks	chips, salty crackers, pretzels, popcorn
Sweets	Milkshakes, yogurt with fruits, ice cream (dairy based) and all ice cream, puddings, frozen yogurt, chocolates, cookies, cakes, plain sugar, pastries, traditional desserts and sweets, jam, chocolate spread, biscuits (plain and sweetened), gello, popsicle
Meat, fish, poultry, eggs	Eggs, beef, fish, poultry
Composite dishes	Meat and poultry-based traditional dishes including soup, legume-based traditional dishes, rice and rice-based dishes, pasta-based dishes
Condiments	Pickles, chicken/meat stock, ground thyme, other condiments
Bread and cereals	Breads, grains, cereals, toast, bread sticks, croissant, bledine cereal, cerelac wheat, corn flakes (plain corn flakes, sweetened corn flakes)
Dairy products	Milk, dairy (cheese, labneh-pressed yogurt) as well as plain yogurt
Pizza pie	Mankouche (Lebanese version of pizza prepared with thyme or cheese), pizza, pies
Tea	Tea
Vegetables and fruits	Vegetables all vegetables and salads, all fruits
Added fats and oils	All added fats and oils including olives oil, olives, peanut butter, tarator (tahineh-based salad dressing)
Starchy food	Starchy vegetables (corn, parsnips, green peas, potato, pumpkin, squash, legumes)
Nuts and seeds	All raw nuts (almonds, walnuts, pistachio nuts, pine nuts)

**Table 6:** Dietary intake by tertiles of scores of the two identified patterns among Lebanese children 2–5 years old (*n* = 525)

Food groups^a^	Fast Food and Sweets pattern		Traditional Lebanese	
	First tertile	Third tertile	First tertile	Third tertile
Sweetened beverage	0.22 ± 0.39	1.51 ± 1.06^c^	1.10 ± 1.13	0.57 ± 0.71^c^
Fast food	0.05 ± 0.14	0.52 ± 0.63^c^	0.31 ± 0.49	0.21 ± 0.38
Salty snacks	0.37 ± 1.10	2.50 ± 2.93^c^	1.84 ± 2.85	1.03 ± 1.86^b^
Sweets	1.50 ± 1.88	4.14 ± 4.01^c^	2.41 ± 2.88	3.16 ± 3.74
Meat, fish, poultry, eggs	0.59 ± 1.04	2.29 ± 3.94^c^	0.79 ± 1.97	1.97 ± 3.96^c^
Pizza and pies	0.11 ± 0.27	0.29 ± 0.60^c^	0.49 ± 0.63	0.04 ± 0.18^c^
Condiments	0.02 ± 0.13	0.62 ± 2.32^c^	0.24 ± 0.97	0.27 ± 1.73
Lebanese traditional dishes	1.35 ± 1.81	0.46 ± 1.05^c^	0.42 ± 0.70	1.19 ± 1.86^c^
Added fats and oils	0.31 ± 0.71	1.55 ± 2.71^c^	0.41 ± 0.92	1.61 ± 2.82^c^
Bread and cereals	1.64 ± 1.40	3.16 ± 2.35^c^	1.35 ± 1.53	4.07 ± 2.61^c^
Dairy products	1.21 ± 1.61	0.84 ± 1.01^b^	0.39 ± 0.62	1.92 ± 1.71^c^
Tea	0.03 ± 0.15	0.30 ± 0.56^c^	0.04 ± 0.16	0.34 ± 0.59^c^
Fruits and vegetables	1.94 ± 1.87	1.65 ± 1.91^c^	0.88 ± 1.06	2.48 ± 2.26^c^
Starchy vegetables and legumes	0.43 ± 1.54	0.22 ± 0.80^c^	0.13 ± 0.48	0.80 ± 1.90^c^
Nuts and seeds	0.58 ± 1.98	0.75 ± 2.92	0.21 ± 1.18	0.94 ± 3.19^b^

^a^Values represent the number of servings consumed per day from each food group

^b^*p* < 0.05

^c^*p* < 0.001
